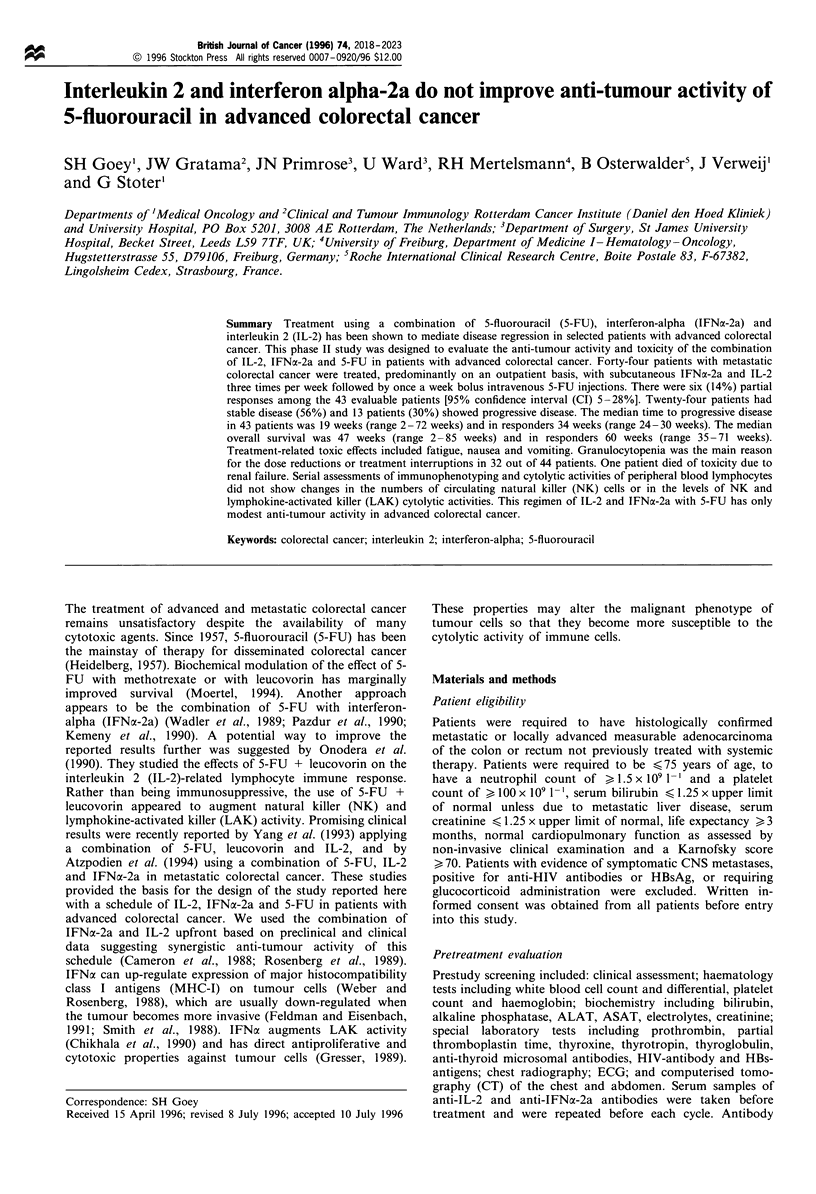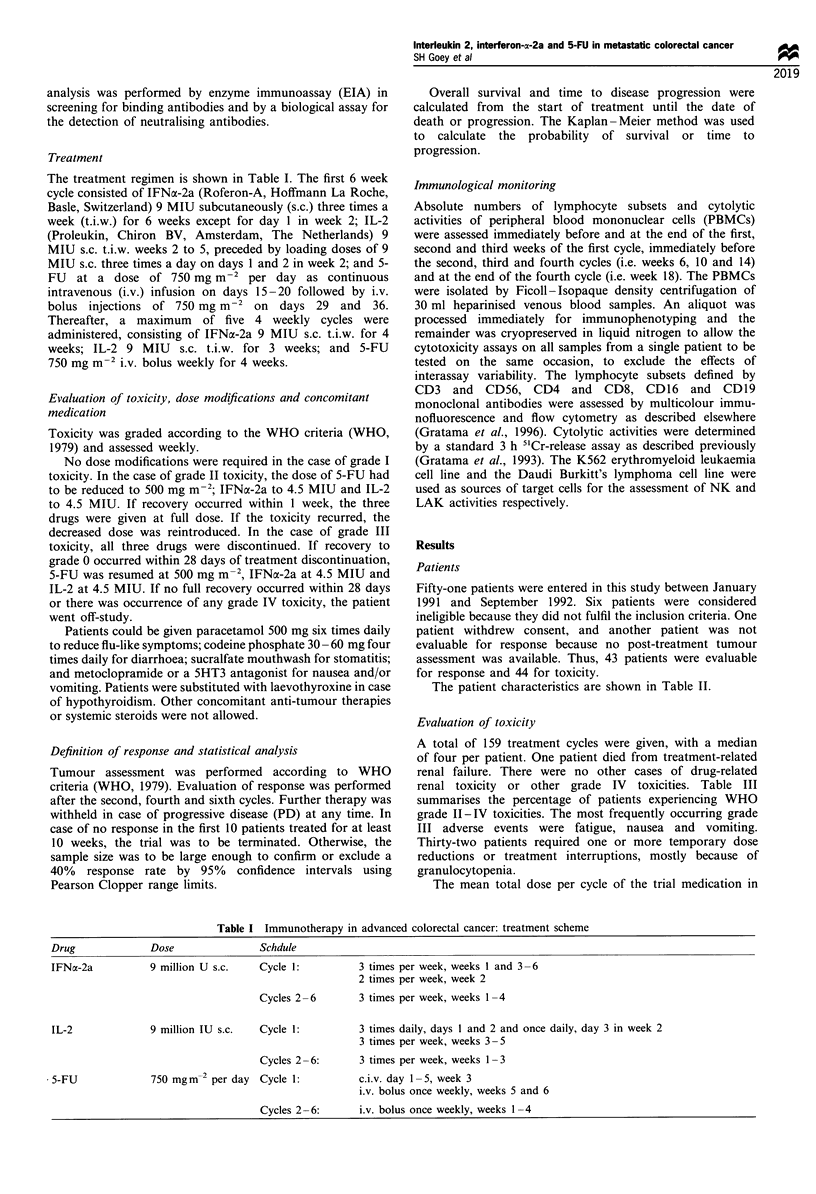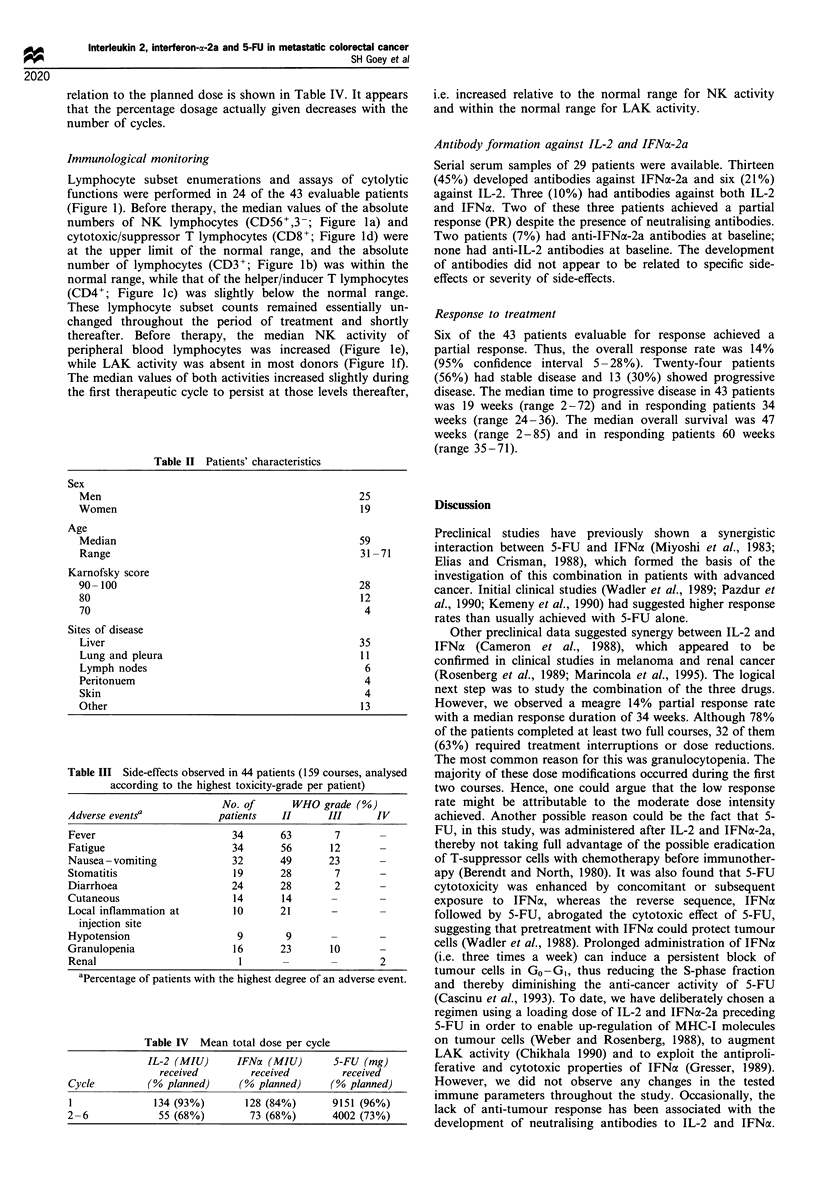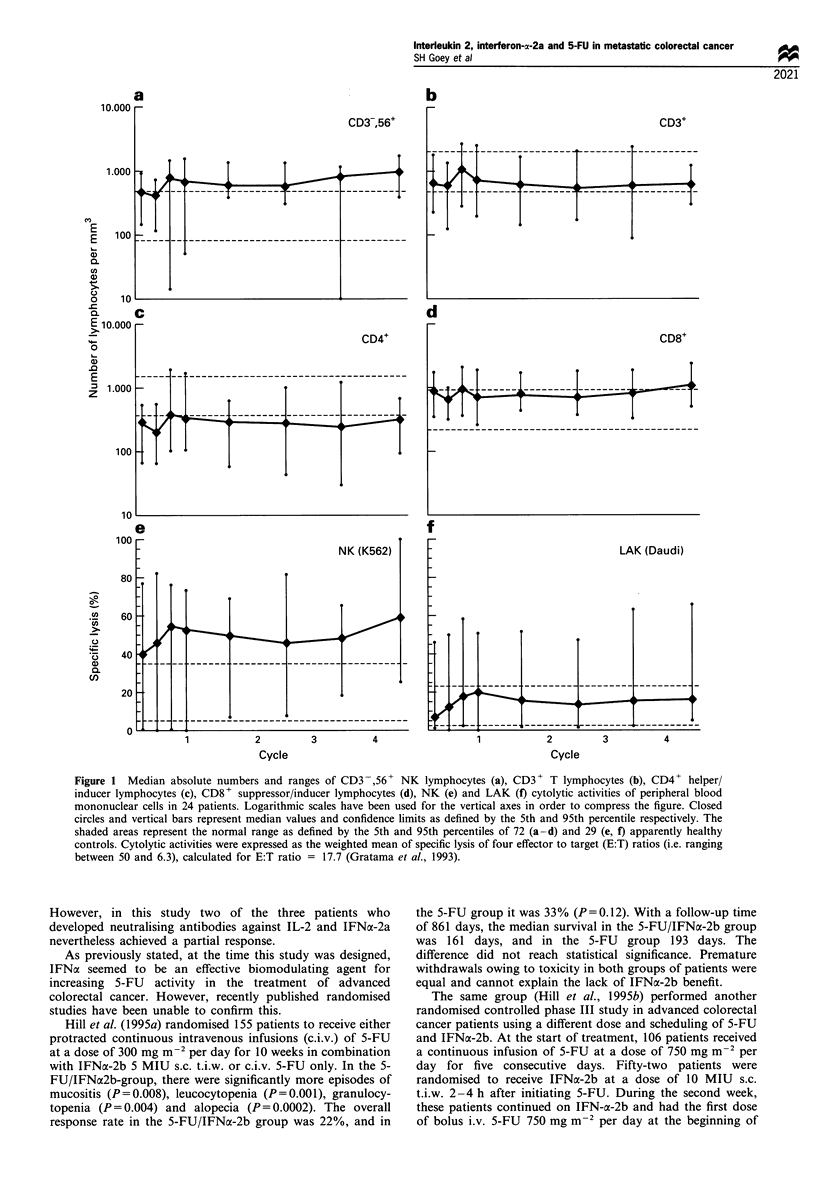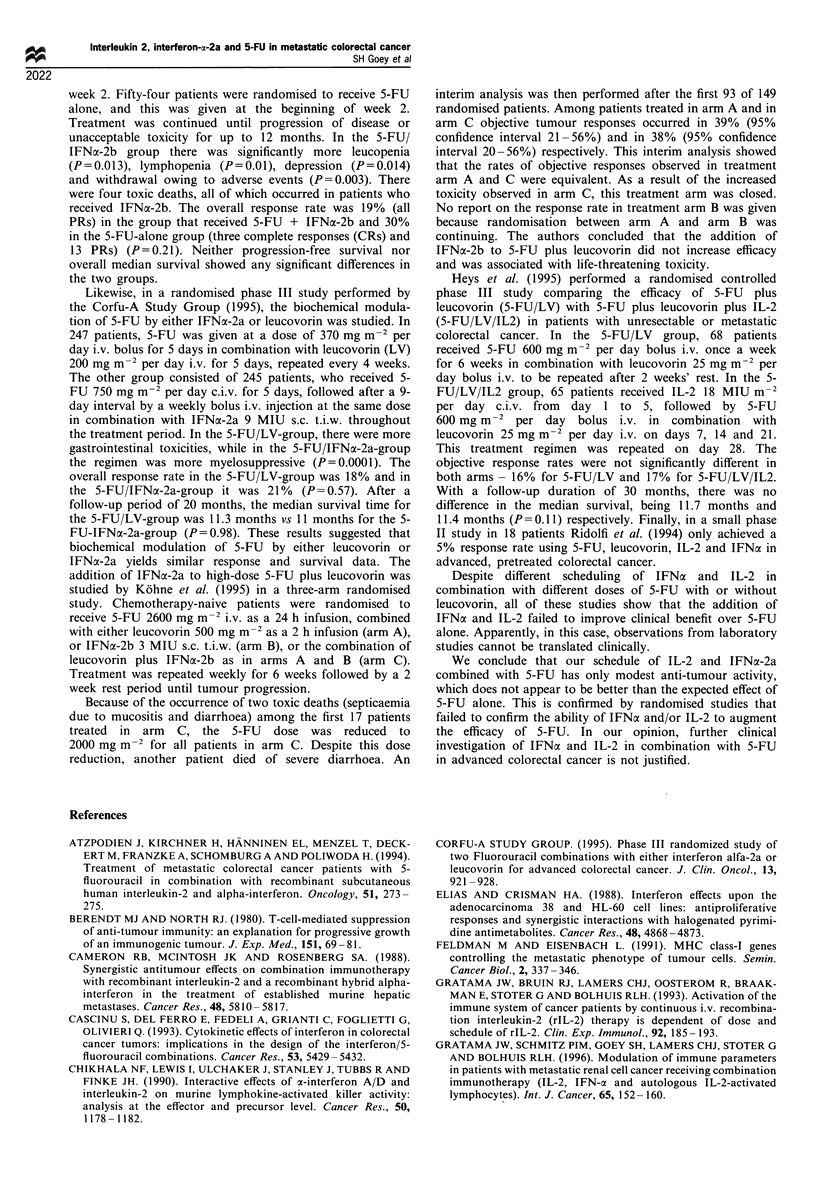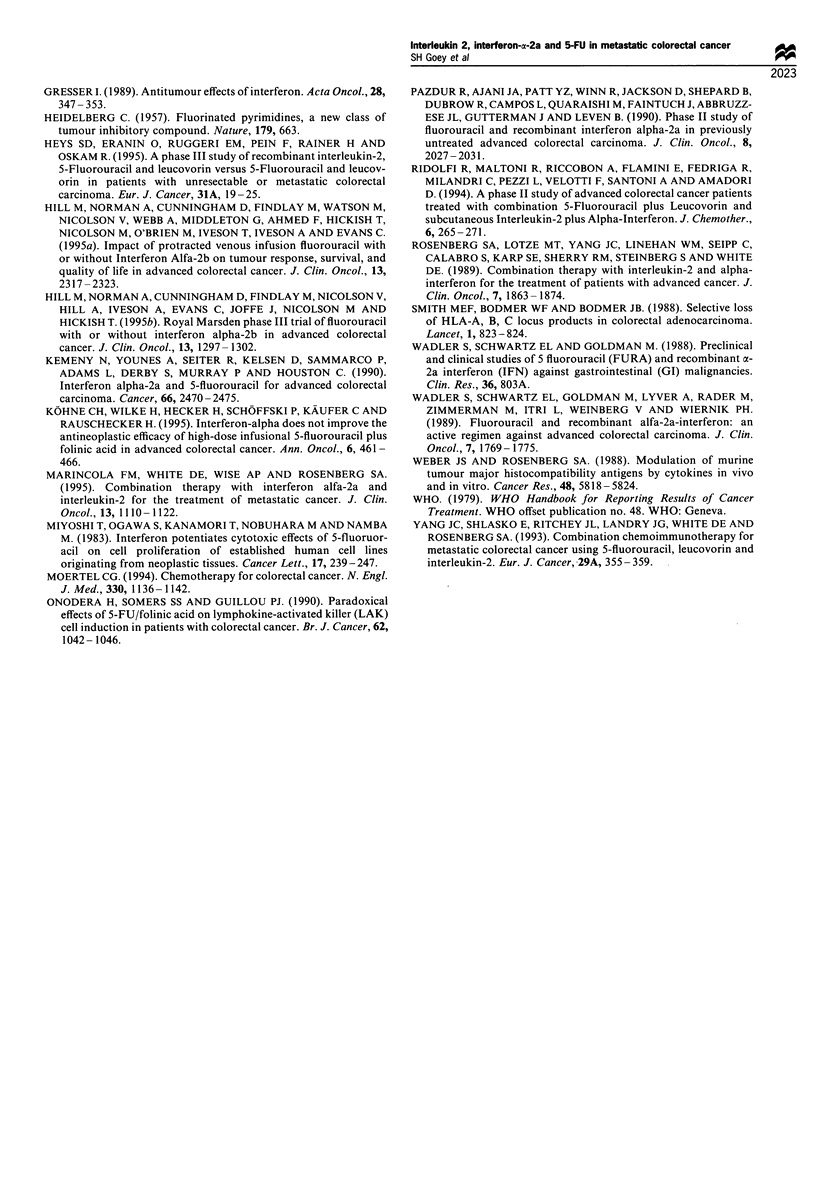# Interleukin 2 and interferon alpha-2a do not improve anti-tumour activity of 5-fluorouracil in advanced colorectal cancer.

**DOI:** 10.1038/bjc.1996.670

**Published:** 1996-12

**Authors:** S. H. Goey, J. W. Gratama, J. N. Primrose, U. Ward, R. H. Mertelsmann, B. Osterwalder, J. Verweij, G. Stoter

**Affiliations:** Department of Medical Oncology, Rotterdam Cancer Institute (Daniel den Hoed Kliniek, The Netherlands.

## Abstract

Treatment using a combination of 5-fluorouracil (5-FU), interferon-alpha (IFN alpha-2a) and interleukin 2 (IL-2) has been shown to mediate disease regression in selected patients with advanced colorectal cancer. This phase II study was designed to evaluate the anti-tumour activity and toxicity of the combination of IL-2, IFN alpha-2a and 5-FU in patients with advanced colorectal cancer. Forty-four patients with metastatic colorectal cancer were treated, predominantly on an outpatient basis, with subcutaneous IFN alpha-2a and IL-2 three times per week followed by once a week bolus intravenous 5-FU injections. There were six (14%) partial responses among the 43 evaluable patients [95% confidence interval (CI) 5-28%]. Twenty-four patients had stable disease (56%) and 13 patients (30%) showed progressive disease. The median time to progressive disease in 43 patients was 19 weeks (range 2-72 weeks) and in responders 34 weeks (range 24-30 weeks). The median overall survival was 47 weeks (range 2-85 weeks) and in responders 60 weeks (range 35-71 weeks). Treatment-related toxic effects included fatigue, nausea and vomiting. Granulocytopenia was the main reason for the dose reductions or treatment interruptions in 32 out of 44 patients. One patient died of toxicity due to renal failure. Serial assessments of immunophenotyping and cytolytic activities of peripheral blood lymphocytes did not show changes in the numbers of circulating natural killer (NK) cells or in the levels of NK and lymphokine-activated killer (LAK) cytolytic activities. This regimen of IL-2 and IFN alpha-2a with 5-FU has only modest anti-tumour activity in advanced colorectal cancer.


British Journal of Cancer (1996) 74, 2018-2023
© 1996 Stockton Press All rights reserved 0007-0920/96 $12.00

### Interleukin 2 and interferon alpha-2a do not improve anti-tumour activity of 5-fluorouracil in advanced colorectal cancer

SH Goey[1], JW Gratama[2], JN Primrose[3], U Ward[3], RH Mertelsmann[4], B Osterwalder[5], J Verweij[1] and G Stoter[1]

*Departments of [1]Medical Oncology and [2]Clinical and Tumour Immunology Rotterdam Cancer Institute (Daniel den Hoed Kliniek) and University Hospital, PO Box 5201, 3008 AE Rotterdam, The Netherlands; [3]Department of Surgery, St James University Hospital, Becket Street, Leeds L59 7TF, UK; [4]University of Freiburg, Department of Medicine I–Hematology–Oncology, Hugstetterstrasse 55, D79106, Freiburg, Germany; [5]Roche International Clinical Research Centre, Boite Postale 83, F-67382, Lingolsheim Cedex, Strasbourg, France.*

**Summary** Treatment using a combination of 5-fluorouracil (5-FU), interferon-alpha (IFNα-2a) and interleukin 2 (IL-2) has been shown to mediate disease regression in selected patients with advanced colorectal cancer. This phase II study was designed to evaluate the anti-tumour activity and toxicity of the combination of IL-2, IFNα-2a and 5-FU in patients with advanced colorectal cancer. Forty-four patients with metastatic colorectal cancer were treated, predominantly on an outpatient basis, with subcutaneous IFNα-2a and IL-2 three times per week followed by once a week bolus intravenous 5-FU injections. There were six (14%) partial responses among the 43 evaluable patients [95% confidence interval (CI) 5–28%]. Twenty-four patients had stable disease (56%) and 13 patients (30%) showed progressive disease. The median time to progressive disease in 43 patients was 19 weeks (range 2–72 weeks) and in responders 34 weeks (range 24–30 weeks). The median overall survival was 47 weeks (range 2–85 weeks) and in responders 60 weeks (range 35–71 weeks). Treatment-related toxic effects included fatigue, nausea and vomiting. Granulocytopenia was the main reason for the dose reductions or treatment interruptions in 32 out of 44 patients. One patient died of toxicity due to renal failure. Serial assessments of immunophenotyping and cytolytic activities of peripheral blood lymphocytes did not show changes in the numbers of circulating natural killer (NK) cells or in the levels of NK and lymphokine-activated killer (LAK) cytolytic activities. This regimen of IL-2 and IFNα-2a with 5-FU has only modest anti-tumour activity in advanced colorectal cancer.

**Keywords:** colorectal cancer; interleukin 2; interferon-alpha; 5-fluorouracil

The treatment of advanced and metastatic colorectal cancer remains unsatisfactory despite the availability of many cytotoxic agents. Since 1957, 5-fluorouracil (5-FU) has been the mainstay of therapy for disseminated colorectal cancer (Heidelberg, 1957). Biochemical modulation of the effect of 5-FU with methotrexate or with leucovorin has marginally improved survival (Moertel, 1994). Another approach appears to be the combination of 5-FU with interferon-alpha (IFNα-2a) (Wadler *et al.*, 1989; Pazdur *et al.*, 1990; Kemeny *et al.*, 1990). A potential way to improve the reported results further was suggested by Onodera *et al.* (1990). They studied the effects of 5-FU + leucovorin on the interleukin 2 (IL-2)-related lymphocyte immune response. Rather than being immunosuppressive, the use of 5-FU + leucovorin appeared to augment natural killer (NK) and lymphokine-activated killer (LAK) activity. Promising clinical results were recently reported by Yang *et al.* (1993) applying a combination of 5-FU, leucovorin and IL-2, and by Atzpodien *et al.* (1994) using a combination of 5-FU, IL-2 and IFNα-2a in metastatic colorectal cancer. These studies provided the basis for the design of the study reported here with a schedule of IL-2, IFNα-2a and 5-FU in patients with advanced colorectal cancer. We used the combination of IFNα-2a and IL-2 upfront based on preclinical and clinical data suggesting synergistic anti-tumour activity of this schedule (Cameron *et al.*, 1988; Rosenberg *et al.*, 1989). IFNα can up-regulate expression of major histocompatibility class I antigens (MHC-I) on tumour cells (Weber and Rosenberg, 1988), which are usually down-regulated when the tumour becomes more invasive (Feldman and Eisenbach, 1991; Smith *et al.*, 1988). IFNα augments LAK activity (Chikhala *et al.*, 1990) and has direct antiproliferative and cytotoxic properties against tumour cells (Gresser, 1989). These properties may alter the malignant phenotype of tumour cells so that they become more susceptible to the cytolytic activity of immune cells.

Correspondence: SH Goey
Received 15 April 1996; revised 8 July 1996; accepted 10 July 1996

### Materials and methods

### *Patient eligibility*

Patients were required to have histologically confirmed metastatic or locally advanced measurable adenocarcinoma of the colon or rectum not previously treated with systemic therapy. Patients were required to be $\leqslant 75$ years of age, to have a neutrophil count of $\geqslant 1.5\times10^9\,l^{-1}$ and a platelet count of $\geqslant 100\times10^9\,l^{-1}$, serum bilirubin $\leqslant 1.25\times$ upper limit of normal unless due to metastatic liver disease, serum creatinine $\leqslant 1.25\times$ upper limit of normal, life expectancy $\geqslant 3$ months, normal cardiopulmonary function as assessed by non-invasive clinical examination and a Karnofsky score $\geqslant 70$. Patients with evidence of symptomatic CNS metastases, positive for anti-HIV antibodies or HBsAg, or requiring glucocorticoid administration were excluded. Written informed consent was obtained from all patients before entry into this study.

### *Pretreatment evaluation*

Prestudy screening included: clinical assessment; haematology tests including white blood cell count and differential, platelet count and haemoglobin; biochemistry including bilirubin, alkaline phosphatase, ALAT, ASAT, electrolytes, creatinine; special laboratory tests including prothrombin, partial thromboplastin time, thyroxine, thyrotropin, thyroglobulin, anti-thyroid microsomal antibodies, HIV-antibody and HBs-antigens; chest radiography; ECG; and computerised tomography (CT) of the chest and abdomen. Serum samples of anti-IL-2 and anti-IFNα-2a antibodies were taken before treatment and were repeated before each cycle. Antibody analysis was performed by enzyme immunoassay (EIA) in screening for binding antibodies and by a biological assay for the detection of neutralising antibodies.

*Treatment*

The treatment regimen is shown in Table I. The first 6 week cycle consisted of IFNα-2a (Roferon-A, Hoffmann La Roche, Basle, Switzerland) 9 MIU subcutaneously (s.c.) three times a week (t.i.w.) for 6 weeks except for day 1 in week 2; IL-2 (Proleukin, Chiron BV, Amsterdam, The Netherlands) 9 MIU s.c. t.i.w. weeks 2 to 5, preceded by loading doses of 9 MIU s.c. three times a day on days 1 and 2 in week 2; and 5-FU at a dose of 750 mg $m^{-2}$ per day as continuous intravenous (i.v.) infusion on days 15–20 followed by i.v. bolus injections of 750 mg $m^{-2}$ on days 29 and 36. Thereafter, a maximum of five 4 weekly cycles were administered, consisting of IFNα-2a 9 MIU s.c. t.i.w. for 4 weeks; IL-2 9 MIU s.c. t.i.w. for 3 weeks; and 5-FU 750 mg $m^{-2}$ i.v. bolus weekly for 4 weeks.

*Evaluation of toxicity, dose modifications and concomitant medication*

Toxicity was graded according to the WHO criteria (WHO, 1979) and assessed weekly.

No dose modifications were required in the case of grade I toxicity. In the case of grade II toxicity, the dose of 5-FU had to be reduced to 500 mg $m^{-2}$; IFNα-2a to 4.5 MIU and IL-2 to 4.5 MIU. If recovery occurred within 1 week, the three drugs were given at full dose. If the toxicity recurred, the decreased dose was reintroduced. In the case of grade III toxicity, all three drugs were discontinued. If recovery to grade 0 occurred within 28 days of treatment discontinuation, 5-FU was resumed at 500 mg $m^{-2}$, IFNα-2a at 4.5 MIU and IL-2 at 4.5 MIU. If no full recovery occurred within 28 days or there was occurrence of any grade IV toxicity, the patient went off-study.

Patients could be given paracetamol 500 mg six times daily to reduce flu-like symptoms; codeine phosphate 30–60 mg four times daily for diarrhoea; sucralfate mouthwash for stomatitis; and metoclopramide or a 5HT3 antagonist for nausea and/or vomiting. Patients were substituted with laevothyroxine in case of hypothyroidism. Other concomitant anti-tumour therapies or systemic steroids were not allowed.

*Definition of response and statistical analysis*

Tumour assessment was performed according to WHO criteria (WHO, 1979). Evaluation of response was performed after the second, fourth and sixth cycles. Further therapy was withheld in case of progressive disease (PD) at any time. In case of no response in the first 10 patients treated for at least 10 weeks, the trial was to be terminated. Otherwise, the sample size was to be large enough to confirm or exclude a 40% response rate by 95% confidence intervals using Pearson Clopper range limits.

Overall survival and time to disease progression were calculated from the start of treatment until the date of death or progression. The Kaplan–Meier method was used to calculate the probability of survival or time to progression.

*Immunological monitoring*

Absolute numbers of lymphocyte subsets and cytolytic activities of peripheral blood mononuclear cells (PBMCs) were assessed immediately before and at the end of the first, second and third weeks of the first cycle, immediately before the second, third and fourth cycles (i.e. weeks 6, 10 and 14) and at the end of the fourth cycle (i.e. week 18). The PBMCs were isolated by Ficoll–Isopaque density centrifugation of 30 ml heparinised venous blood samples. An aliquot was processed immediately for immunophenotyping and the remainder was cryopreserved in liquid nitrogen to allow the cytotoxicity assays on all samples from a single patient to be tested on the same occasion, to exclude the effects of interassay variability. The lymphocyte subsets defined by CD3 and CD56, CD4 and CD8, CD16 and CD19 monoclonal antibodies were assessed by multicolour immunofluorescence and flow cytometry as described elsewhere (Gratama *et al*., 1996). Cytolytic activities were determined by a standard 3 h $^{51}$Cr-release assay as described previously (Gratama *et al*., 1993). The K562 erythromyeloid leukaemia cell line and the Daudi Burkitt's lymphoma cell line were used as sources of target cells for the assessment of NK and LAK activities respectively.

### Results

*Patients*

Fifty-one patients were entered in this study between January 1991 and September 1992. Six patients were considered ineligible because they did not fulfil the inclusion criteria. One patient withdrew consent, and another patient was not evaluable for response because no post-treatment tumour assessment was available. Thus, 43 patients were evaluable for response and 44 for toxicity.

The patient characteristics are shown in Table II.

*Evaluation of toxicity*

A total of 159 treatment cycles were given, with a median of four per patient. One patient died from treatment-related renal failure. There were no other cases of drug-related renal toxicity or other grade IV toxicities. Table III summarises the percentage of patients experiencing WHO grade II–IV toxicities. The most frequently occurring grade III adverse events were fatigue, nausea and vomiting. Thirty-two patients required one or more temporary dose reductions or treatment interruptions, mostly because of granulocytopenia.

The mean total dose per cycle of the trial medication in

**Table I** Immunotherapy in advanced colorectal cancer: treatment scheme

| *Drug* | *Dose* | *Schdule* | |
|---|---|---|---|
| IFNα-2a | 9 million U s.c. | Cycle 1: | 3 times per week, weeks 1 and 3–6<br>2 times per week, week 2 |
| | | Cycles 2–6 | 3 times per week, weeks 1–4 |
| IL-2 | 9 million IU s.c. | Cycle 1: | 3 times daily, days 1 and 2 and once daily, day 3 in week 2<br>3 times per week, weeks 3–5 |
| | | Cycles 2–6: | 3 times per week, weeks 1–3 |
| 5-FU | 750 mg $m^{-2}$ per day | Cycle 1: | c.i.v. day 1–5, week 3<br>i.v. bolus once weekly, weeks 5 and 6 |
| | | Cycles 2–6: | i.v. bolus once weekly, weeks 1–4 |

relation to the planned dose is shown in Table IV. It appears that the percentage dosage actually given decreases with the number of cycles.

*Immunological monitoring*

Lymphocyte subset enumerations and assays of cytolytic functions were performed in 24 of the 43 evaluable patients (Figure 1). Before therapy, the median values of the absolute numbers of NK lymphocytes ($CD56^+,3^-$; Figure 1a) and cytotoxic/suppressor T lymphocytes ($CD8^+$; Figure 1d) were at the upper limit of the normal range, and the absolute number of lymphocytes ($CD3^+$; Figure 1b) was within the normal range, while that of the helper/inducer T lymphocytes ($CD4^+$; Figure 1c) was slightly below the normal range. These lymphocyte subset counts remained essentially unchanged throughout the period of treatment and shortly thereafter. Before therapy, the median NK activity of peripheral blood lymphocytes was increased (Figure 1e), while LAK activity was absent in most donors (Figure 1f). The median values of both activities increased slightly during the first therapeutic cycle to persist at those levels thereafter, i.e. increased relative to the normal range for NK activity and within the normal range for LAK activity.

*Antibody formation against IL-2 and IFNα-2a*

Serial serum samples of 29 patients were available. Thirteen (45%) developed antibodies against IFNα-2a and six (21%) against IL-2. Three (10%) had antibodies against both IL-2 and IFNα. Two of these three patients achieved a partial response (PR) despite the presence of neutralising antibodies. Two patients (7%) had anti-IFNα-2a antibodies at baseline; none had anti-IL-2 antibodies at baseline. The development of antibodies did not appear to be related to specific side-effects or severity of side-effects.

*Response to treatment*

Six of the 43 patients evaluable for response achieved a partial response. Thus, the overall response rate was 14% (95% confidence interval 5–28%). Twenty-four patients (56%) had stable disease and 13 (30%) showed progressive disease. The median time to progressive disease in 43 patients was 19 weeks (range 2–72) and in responding patients 34 weeks (range 24–36). The median overall survival was 47 weeks (range 2–85) and in responding patients 60 weeks (range 35–71).

**Table II** Patients' characteristics

| | |
|---|---|
| Sex | |
| Men | 25 |
| Women | 19 |
| Age | |
| Median | 59 |
| Range | 31–71 |
| Karnofsky score | |
| 90–100 | 28 |
| 80 | 12 |
| 70 | 4 |
| Sites of disease | |
| Liver | 35 |
| Lung and pleura | 11 |
| Lymph nodes | 6 |
| Peritonuem | 4 |
| Skin | 4 |
| Other | 13 |

**Table III** Side-effects observed in 44 patients (159 courses, analysed according to the highest toxicity-grade per patient)

| *Adverse events*[a] | *No. of patients* | *WHO grade (%)* *II* | *III* | *IV* |
|---|---|---|---|---|
| Fever | 34 | 63 | 7 | – |
| Fatigue | 34 | 56 | 12 | – |
| Nausea–vomiting | 32 | 49 | 23 | – |
| Stomatitis | 19 | 28 | 7 | – |
| Diarrhoea | 24 | 28 | 2 | – |
| Cutaneous | 14 | 14 | – | – |
| Local inflammation at injection site | 10 | 21 | – | – |
| Hypotension | 9 | 9 | – | – |
| Granulopenia | 16 | 23 | 10 | – |
| Renal | 1 | – | – | 2 |

[a]Percentage of patients with the highest degree of an adverse event.

**Table IV** Mean total dose per cycle

| *Cycle* | *IL-2 (MIU) received (% planned)* | *IFNα (MIU) received (% planned)* | *5-FU (mg) received (% planned)* |
|---|---|---|---|
| 1 | 134 (93%) | 128 (84%) | 9151 (96%) |
| 2–6 | 55 (68%) | 73 (68%) | 4002 (73%) |

### Discussion

Preclinical studies have previously shown a synergistic interaction between 5-FU and IFNα (Miyoshi *et al.*, 1983; Elias and Crisman, 1988), which formed the basis of the investigation of this combination in patients with advanced cancer. Initial clinical studies (Wadler *et al.*, 1989; Pazdur *et al.*, 1990; Kemeny *et al.*, 1990) had suggested higher response rates than usually achieved with 5-FU alone.

Other preclinical data suggested synergy between IL-2 and IFNα (Cameron *et al.*, 1988), which appeared to be confirmed in clinical studies in melanoma and renal cancer (Rosenberg *et al.*, 1989; Marincola *et al.*, 1995). The logical next step was to study the combination of the three drugs. However, we observed a meagre 14% partial response rate with a median response duration of 34 weeks. Although 78% of the patients completed at least two full courses, 32 of them (63%) required treatment interruptions or dose reductions. The most common reason for this was granulocytopenia. The majority of these dose modifications occurred during the first two courses. Hence, one could argue that the low response rate might be attributable to the moderate dose intensity achieved. Another possible reason could be the fact that 5-FU, in this study, was administered after IL-2 and IFNα-2a, thereby not taking full advantage of the possible eradication of T-suppressor cells with chemotherapy before immunotherapy (Berendt and North, 1980). It was also found that 5-FU cytotoxicity was enhanced by concomitant or subsequent exposure to IFNα, whereas the reverse sequence, IFNα followed by 5-FU, abrogated the cytotoxic effect of 5-FU, suggesting that pretreatment with IFNα could protect tumour cells (Wadler *et al.*, 1988). Prolonged administration of IFNα (i.e. three times a week) can induce a persistent block of tumour cells in $G_0-G_1$, thus reducing the S-phase fraction and thereby diminishing the anti-cancer activity of 5-FU (Cascinu *et al.*, 1993). To date, we have deliberately chosen a regimen using a loading dose of IL-2 and IFNα-2a preceding 5-FU in order to enable up-regulation of MHC-I molecules on tumour cells (Weber and Rosenberg, 1988), to augment LAK activity (Chikhala 1990) and to exploit the antiproliferative and cytotoxic properties of IFNα (Gresser, 1989). However, we did not observe any changes in the tested immune parameters throughout the study. Occasionally, the lack of anti-tumour response has been associated with the development of neutralising antibodies to IL-2 and IFNα.

**Figure 1** Median absolute numbers and ranges of $CD3^-,56^+$ NK lymphocytes (**a**), $CD3^+$ T lymphocytes (**b**), $CD4^+$ helper/inducer lymphocytes (**c**), $CD8^+$ suppressor/inducer lymphocytes (**d**), NK (**e**) and LAK (**f**) cytolytic activities of peripheral blood mononuclear cells in 24 patients. Logarithmic scales have been used for the vertical axes in order to compress the figure. Closed circles and vertical bars represent median values and confidence limits as defined by the 5th and 95th percentile respectively. The shaded areas represent the normal range as defined by the 5th and 95th percentiles of 72 (**a**–**d**) and 29 (**e**, **f**) apparently healthy controls. Cytolytic activities were expressed as the weighted mean of specific lysis of four effector to target (E:T) ratios (i.e. ranging between 50 and 6.3), calculated for E:T ratio = 17.7 (Gratama *et al*., 1993).

However, in this study two of the three patients who developed neutralising antibodies against IL-2 and IFNα-2a nevertheless achieved a partial response.

As previously stated, at the time this study was designed, IFNα seemed to be an effective biomodulating agent for increasing 5-FU activity in the treatment of advanced colorectal cancer. However, recently published randomised studies have been unable to confirm this.

Hill *et al.* (1995*a*) randomised 155 patients to receive either protracted continuous intravenous infusions (c.i.v.) of 5-FU at a dose of 300 mg $m^{-2}$ per day for 10 weeks in combination with IFNα-2b 5 MIU s.c. t.i.w. or c.i.v. 5-FU only. In the 5-FU/IFNα2b-group, there were significantly more episodes of mucositis ($P$=0.008), leucocytopenia ($P$=0.001), granulocytopenia ($P$=0.004) and alopecia ($P$=0.0002). The overall response rate in the 5-FU/IFNα-2b group was 22%, and in the 5-FU group it was 33% ($P$=0.12). With a follow-up time of 861 days, the median survival in the 5-FU/IFNα-2b group was 161 days, and in the 5-FU group 193 days. The difference did not reach statistical significance. Premature withdrawals owing to toxicity in both groups of patients were equal and cannot explain the lack of IFNα-2b benefit.

The same group (Hill *et al.*, 1995*b*) performed another randomised controlled phase III study in advanced colorectal cancer patients using a different dose and scheduling of 5-FU and IFNα-2b. At the start of treatment, 106 patients received a continuous infusion of 5-FU at a dose of 750 mg $m^{-2}$ per day for five consecutive days. Fifty-two patients were randomised to receive IFNα-2b at a dose of 10 MIU s.c. t.i.w. 2–4 h after initiating 5-FU. During the second week, these patients continued on IFN-α-2b and had the first dose of bolus i.v. 5-FU 750 mg $m^{-2}$ per day at the beginning of week 2. Fifty-four patients were randomised to receive 5-FU alone, and this was given at the beginning of week 2. Treatment was continued until progression of disease or unacceptable toxicity for up to 12 months. In the 5-FU/IFNα-2b group there was significantly more leucopenia ($P=0.013$), lymphopenia ($P=0.01$), depression ($P=0.014$) and withdrawal owing to adverse events ($P=0.003$). There were four toxic deaths, all of which occurred in patients who received IFNα-2b. The overall response rate was 19% (all PRs) in the group that received 5-FU + IFNα-2b and 30% in the 5-FU-alone group (three complete responses (CRs) and 13 PRs) ($P=0.21$). Neither progression-free survival nor overall median survival showed any significant differences in the two groups.

Likewise, in a randomised phase III study performed by the Corfu-A Study Group (1995), the biochemical modulation of 5-FU by either IFNα-2a or leucovorin was studied. In 247 patients, 5-FU was given at a dose of 370 mg $m^{-2}$ per day i.v. bolus for 5 days in combination with leucovorin (LV) 200 mg $m^{-2}$ per day i.v. for 5 days, repeated every 4 weeks. The other group consisted of 245 patients, who received 5-FU 750 mg $m^{-2}$ per day c.i.v. for 5 days, followed after a 9-day interval by a weekly bolus i.v. injection at the same dose in combination with IFNα-2a 9 MIU s.c. t.i.w. throughout the treatment period. In the 5-FU/LV-group, there were more gastrointestinal toxicities, while in the 5-FU/IFNα-2a-group the regimen was more myelosuppressive ($P=0.0001$). The overall response rate in the 5-FU/LV-group was 18% and in the 5-FU/IFNα-2a-group it was 21% ($P=0.57$). After a follow-up period of 20 months, the median survival time for the 5-FU/LV-group was 11.3 months *vs* 11 months for the 5-FU-IFNα-2a-group ($P=0.98$). These results suggested that biochemical modulation of 5-FU by either leucovorin or IFNα-2a yields similar response and survival data. The addition of IFNα-2a to high-dose 5-FU plus leucovorin was studied by Köhne *et al.* (1995) in a three-arm randomised study. Chemotherapy-naive patients were randomised to receive 5-FU 2600 mg $m^{-2}$ i.v. as a 24 h infusion, combined with either leucovorin 500 mg $m^{-2}$ as a 2 h infusion (arm A), or IFNα-2b 3 MIU s.c. t.i.w. (arm B), or the combination of leucovorin plus IFNα-2b as in arms A and B (arm C). Treatment was repeated weekly for 6 weeks followed by a 2 week rest period until tumour progression.

Because of the occurrence of two toxic deaths (septicaemia due to mucositis and diarrhoea) among the first 17 patients treated in arm C, the 5-FU dose was reduced to 2000 mg $m^{-2}$ for all patients in arm C. Despite this dose reduction, another patient died of severe diarrhoea. An interim analysis was then performed after the first 93 of 149 randomised patients. Among patients treated in arm A and in arm C objective tumour responses occurred in 39% (95% confidence interval 21–56%) and in 38% (95% confidence interval 20–56%) respectively. This interim analysis showed that the rates of objective responses observed in treatment arm A and C were equivalent. As a result of the increased toxicity observed in arm C, this treatment arm was closed. No report on the response rate in treatment arm B was given because randomisation between arm A and arm B was continuing. The authors concluded that the addition of IFNα-2b to 5-FU plus leucovorin did not increase efficacy and was associated with life-threatening toxicity.

Heys *et al.* (1995) performed a randomised controlled phase III study comparing the efficacy of 5-FU plus leucovorin (5-FU/LV) with 5-FU plus leucovorin plus IL-2 (5-FU/LV/IL2) in patients with unresectable or metastatic colorectal cancer. In the 5-FU/LV group, 68 patients received 5-FU 600 mg $m^{-2}$ per day bolus i.v. once a week for 6 weeks in combination with leucovorin 25 mg $m^{-2}$ per day bolus i.v. to be repeated after 2 weeks' rest. In the 5-FU/LV/IL2 group, 65 patients received IL-2 18 MIU $m^{-2}$ per day c.i.v. from day 1 to 5, followed by 5-FU 600 mg $m^{-2}$ per day bolus i.v. in combination with leucovorin 25 mg $m^{-2}$ per day i.v. on days 7, 14 and 21. This treatment regimen was repeated on day 28. The objective response rates were not significantly different in both arms – 16% for 5-FU/LV and 17% for 5-FU/LV/IL2. With a follow-up duration of 30 months, there was no difference in the median survival, being 11.7 months and 11.4 months ($P=0.11$) respectively. Finally, in a small phase II study in 18 patients Ridolfi *et al.* (1994) only achieved a 5% response rate using 5-FU, leucovorin, IL-2 and IFNα in advanced, pretreated colorectal cancer.

Despite different scheduling of IFNα and IL-2 in combination with different doses of 5-FU with or without leucovorin, all of these studies show that the addition of IFNα and IL-2 failed to improve clinical benefit over 5-FU alone. Apparently, in this case, observations from laboratory studies cannot be translated clinically.

We conclude that our schedule of IL-2 and IFNα-2a combined with 5-FU has only modest anti-tumour activity, which does not appear to be better than the expected effect of 5-FU alone. This is confirmed by randomised studies that failed to confirm the ability of IFNα and/or IL-2 to augment the efficacy of 5-FU. In our opinion, further clinical investigation of IFNα and IL-2 in combination with 5-FU in advanced colorectal cancer is not justified.

### References

ATZPODIEN J, KIRCHNER H, HÄNNINEN EL, MENZEL T, DECKERT M, FRANZKE A, SCHOMBURG A AND POLIWODA H. (1994). Treatment of metastatic colorectal cancer patients with 5-fluorouracil in combination with recombinant subcutaneous human interleukin-2 and alpha-interferon. *Oncology*, **51**, 273–275.

BERENDT MJ AND NORTH RJ. (1980). T-cell-mediated suppression of anti-tumour immunity: an explanation for progressive growth of an immunogenic tumour. *J. Exp. Med.*, **151**, 69–81.

CAMERON RB, MCINTOSH JK AND ROSENBERG SA. (1988). Synergistic antitumour effects on combination immunotherapy with recombinant interleukin-2 and a recombinant hybrid alpha-interferon in the treatment of established murine hepatic metastases. *Cancer Res.*, **48**, 5810–5817.

CASCINU S, DEL FERRO E, FEDELI A, GRIANTI C, FOGLIETTI G, OLIVIERI Q. (1993). Cytokinetic effects of interferon in colorectal cancer tumors: implications in the design of the interferon/5-fluorouracil combinations. *Cancer Res.*, **53**, 5429–5432.

CHIKHALA NF, LEWIS I, ULCHAKER J, STANLEY J, TUBBS R AND FINKE JH. (1990). Interactive effects of α-interferon A/D and interleukin-2 on murine lymphokine-activated killer activity: analysis at the effector and precursor level. *Cancer Res.*, **50**, 1178–1182.

CORFU-A STUDY GROUP. (1995). Phase III randomized study of two Fluorouracil combinations with either interferon alfa-2a or leucovorin for advanced colorectal cancer. *J. Clin. Oncol.*, **13**, 921–928.

ELIAS AND CRISMAN HA. (1988). Interferon effects upon the adenocarcinoma 38 and HL-60 cell lines: antiproliferative responses and synergistic interactions with halogenated pyrimidine antimetabolites. *Cancer Res.*, **48**, 4868–4873.

FELDMAN M AND EISENBACH L. (1991). MHC class-I genes controlling the metastatic phenotype of tumour cells. *Semin. Cancer Biol.*, **2**, 337–346.

GRATAMA JW, BRUIN RJ, LAMERS CHJ, OOSTEROM R, BRAAKMAN E, STOTER G AND BOLHUIS RLH. (1993). Activation of the immune system of cancer patients by continuous i.v. recombination interleukin-2 (rIL-2) therapy is dependent of dose and schedule of rIL-2. *Clin. Exp. Immunol.*, **92**, 185–193.

GRATAMA JW, SCHMITZ PIM, GOEY SH, LAMERS CHJ, STOTER G AND BOLHUIS RLH. (1996). Modulation of immune parameters in patients with metastatic renal cell cancer receiving combination immunotherapy (IL-2, IFN-α and autologous IL-2-activated lymphocytes). *Int. J. Cancer*, **65**, 152–160.

GRESSER I. (1989). Antitumour effects of interferon. *Acta Oncol.*, **28,** 347–353.

HEIDELBERG C. (1957). Fluorinated pyrimidines, a new class of tumour inhibitory compound. *Nature*, **179,** 663.

HEYS SD, ERANIN O, RUGGERI EM, PEIN F, RAINER H AND OSKAM R. (1995). A phase III study of recombinant interleukin-2, 5-Fluorouracil and leucovorin versus 5-Fluorouracil and leucovorin in patients with unresectable or metastatic colorectal carcinoma. *Eur. J. Cancer*, **31A**, 19–25.

HILL M, NORMAN A, CUNNINGHAM D, FINDLAY M, WATSON M, NICOLSON V, WEBB A, MIDDLETON G, AHMED F, HICKISH T, NICOLSON M, O'BRIEN M, IVESON T, IVESON A AND EVANS C. (1995*a*). Impact of protracted venous infusion fluorouracil with or without Interferon Alfa-2b on tumour response, survival, and quality of life in advanced colorectal cancer. *J. Clin. Oncol.*, **13,** 2317–2323.

HILL M, NORMAN A, CUNNINGHAM D, FINDLAY M, NICOLSON V, HILL A, IVESON A, EVANS C, JOFFE J, NICOLSON M AND HICKISH T. (1995*b*). Royal Marsden phase III trial of fluorouracil with or without interferon alpha-2b in advanced colorectal cancer. *J. Clin. Oncol.*, **13,** 1297–1302.

KEMENY N, YOUNES A, SEITER R, KELSEN D, SAMMARCO P, ADAMS L, DERBY S, MURRAY P AND HOUSTON C. (1990). Interferon alpha-2a and 5-fluorouracil for advanced colorectal carcinoma. *Cancer*, **66,** 2470–2475.

KÖHNE CH, WILKE H, HECKER H, SCHÖFFSKI P, KÄUFER C AND RAUSCHECKER H. (1995). Interferon-alpha does not improve the antineoplastic efficacy of high-dose infusional 5-fluorouracil plus folinic acid in advanced colorectal cancer. *Ann. Oncol.*, **6**, 461–466.

MARINCOLA FM, WHITE DE, WISE AP AND ROSENBERG SA. (1995). Combination therapy with interferon alfa-2a and interleukin-2 for the treatment of metastatic cancer. *J. Clin. Oncol.*, **13,** 1110–1122.

MIYOSHI T, OGAWA S, KANAMORI T, NOBUHARA M AND NAMBA M. (1983). Interferon potentiates cytotoxic effects of 5-fluorouracil on cell proliferation of established human cell lines originating from neoplastic tissues. *Cancer Lett.*, **17,** 239–247.

MOERTEL CG. (1994). Chemotherapy for colorectal cancer. *N. Engl. J. Med.*, **330,** 1136–1142.

ONODERA H, SOMERS SS AND GUILLOU PJ. (1990). Paradoxical effects of 5-FU/folinic acid on lymphokine-activated killer (LAK) cell induction in patients with colorectal cancer. *Br. J. Cancer*, **62,** 1042–1046.

PAZDUR R, AJANI JA, PATT YZ, WINN R, JACKSON D, SHEPARD B, DUBROW R, CAMPOS L, QUARAISHI M, FAINTUCH J, ABBRUZZESE JL, GUTTERMAN J AND LEVEN B. (1990). Phase II study of fluorouracil and recombinant interferon alpha-2a in previously untreated advanced colorectal carcinoma. *J. Clin. Oncol.*, **8,** 2027–2031.

RIDOLFI R, MALTONI R, RICCOBON A, FLAMINI E, FEDRIGA R, MILANDRI C, PEZZI L, VELOTTI F, SANTONI A AND AMADORI D. (1994). A phase II study of advanced colorectal cancer patients treated with combination 5-Fluorouracil plus Leucovorin and subcutaneous Interleukin-2 plus Alpha-Interferon. *J. Chemother.*, **6,** 265–271.

ROSENBERG SA, LOTZE MT, YANG JC, LINEHAN WM, SEIPP C, CALABRO S, KARP SE, SHERRY RM, STEINBERG S AND WHITE DE. (1989). Combination therapy with interleukin-2 and alpha-interferon for the treatment of patients with advanced cancer. *J. Clin. Oncol.*, **7,** 1863–1874.

SMITH MEF, BODMER WF AND BODMER JB. (1988). Selective loss of HLA-A, B, C locus products in colorectal adenocarcinoma. *Lancet*, **1,** 823–824.

WADLER S, SCHWARTZ EL AND GOLDMAN M. (1988). Preclinical and clinical studies of 5 fluorouracil (FURA) and recombinant α-2a interferon (IFN) against gastrointestinal (GI) malignancies. *Clin. Res.*, **36,** 803A.

WADLER S, SCHWARTZ EL, GOLDMAN M, LYVER A, RADER M, ZIMMERMAN M, ITRI L, WEINBERG V AND WIERNIK PH. (1989). Fluorouracil and recombinant alfa-2a-interferon: an active regimen against advanced colorectal carcinoma. *J. Clin. Oncol.*, **7,** 1769–1775.

WEBER JS AND ROSENBERG SA. (1988). Modulation of murine tumour major histocompatibility antigens by cytokines in vivo and in vitro. *Cancer Res.*, **48,** 5818–5824.

WHO. (1979). *WHO Handbook for Reporting Results of Cancer Treatment.* WHO offset publication no. 48. WHO: Geneva.

YANG JC, SHLASKO E, RITCHEY JL, LANDRY JG, WHITE DE AND ROSENBERG SA. (1993). Combination chemoimmunotherapy for metastatic colorectal cancer using 5-fluorouracil, leucovorin and interleukin-2. *Eur. J. Cancer*, **29A,** 355–359.